# Systematic review of economic evaluations investigating education, exercise, and dietary weight management to manage hip and knee osteoarthritis: protocol

**DOI:** 10.1186/s13643-020-01492-6

**Published:** 2020-10-06

**Authors:** Darren R. Mazzei, Ayoola Ademola, J. Haxby Abbott, Tolulpe Sajobi, Kevin Hildebrand, Deborah A. Marshall

**Affiliations:** 1 McCaig Institute for Bone and Joint Health and Department of Community Health Sciences, Cumming School of Medicine, University of Calgary, 3280 Hospital Drive NW, Calgary, Alberta T2N 4Z6 Canada; 2 Department of Surgical Sciences, University of Otago, 201 Great King Street, Dunedin, Otago 9016 New Zealand; 3 McCaig Institute for Bone and Joint Health and Department of Surgery, University of Calgary, 1403 29 St NW, Calgary, Alberta T2N 2T9 Canada

**Keywords:** Osteoarthritis, Hip osteoarthritis, Knee osteoarthritis, Education, Patient education, Health education, Exercise, Exercise therapy, Diet, Weight loss, Diet therapy, Cost-effectiveness analysis, Cost-utility analysis, Cost-benefit analysis

## Abstract

**Background:**

International guidelines recommend education, exercise, and dietary weight management as core treatments to manage osteoarthritis (OA) regardless of disease severity or co-morbidity. Evidence supports the clinical effectiveness of OA management programs, but the cost-effectiveness of core treatments remains unclear. We will systematically review, synthesize, and assess the literature in economic evaluations of core treatments (education, exercise, and dietary weight management) for the management of hip and/or knee OA.

**Methods:**

We will search the following elecftronic databases (from inception onwards): MEDLINE, EMBASE, Cochrane Central Register of Controlled Trials (CENTRAL), National Health Services Economic Evaluation Database, and EconLit. Economic evaluations alongside randomized or nonrandomized clinical trials investigating OA education, exercise, and dietary weight management interventions will be included. Title, abstract, and full text of relevant publications will be screened independently by two reviewers. A content matter expert will resolve any conflicts between two reviewers. Key information from relevant papers will be extracted and tabulated to provide an overview of the published literature. Methodological quality will be evaluated using the Consensus on Health Economic Criteria list. A narrative synthesis without meta-analysis will be conducted. Subgroup analysis will attempt to find trends between research methods, intervention characteristics, and results.

**Discussion:**

The findings of this review will evaluate the breadth and quality of economic evaluations conducted alongside clinical trials for core treatments in OA management.

**Systematic review registration:**

PROSPERO CRD42020155964

## Background

Osteoarthritis (OA) is a major cause of disability [1]. International guidelines recommend education, exercise, and dietary weight management (if appropriate) as the core treatments for OA before progressing to pharmacological and surgical interventions [2]. However, low uptake of core treatments persists [3]. To improve uptake, various OA management programs (OAMPs) using core treatments have been developed, evaluated, and shown to be clinically effective, but consensus has not been reached on the cost-effectiveness of these programs [4]. Evaluating cost-effectiveness of OAMPs has been identified as a research priority by a group of international OA researchers [5]. A cost-effective intervention means the treatment provides more health benefit at an additional cost but within the decisions-makers’ willingness to pay for those health benefits [6]. Cost-effectiveness is an important consideration for the implementation of OAMPs in budget-constrained health care systems [6].

A 2012 systematic review found sparse literature and recommended more studies that evaluate the cost-effectiveness of nonsurgical and nonpharmacological interventions in OA [7]. Authors found eleven publications showing mixed results and a high a risk of bias due to poor quality methodology [7]. To improve study quality, the authors recommended standardized methodologies such as generic health outcome measures, capturing all disease-related costs and comparing the intervention to usual care [7]. Since 2012, methodological recommendations [8–11] and reporting criteria [12, 13] for economic evaluations have been produced. To identify research gaps, a systematic review identifying the current body of empirical knowledge is warranted.

Economic evaluations inform decision-makers about the consequences of resource allocation decisions by comparing the cost and health outcomes of two or more interventions [6]. Clinical trials can be used as a vehicle to conduct economic evaluations by collecting patient-level costs and health outcomes [6]. Alternatively, the relevant literature can be synthesized using decision-analytic modeling techniques to produce economic estimates [6]. Methodological differences between model and trial-based economic evaluations limit their comparability so authors have argued to systematically review these distinct methodologies separately [14]. Different quality assessment tools have also been published to evaluate trial and model-based economic evaluations separately [15]. Lastly, trial-based economic evaluations are more common in the research field evaluating nonpharmacological and nonsurgical treatment of OA [9]. For these reasons our systematic review will focus on economic studies where primary data was collected. We will synthesize and assess quality of economic evaluations investigating the cost-effectiveness of core treatments (education, exercise, and dietary weight management) for the management of hip and/or knee OA.

### Review question

Are core treatments (exercise, education, and dietary weight management) for the management of hip and knee OA considered cost-effective in comparison to usual care or controls in different health care systems and perspectives?

## Methods

This review protocol is being reported in accordance with the reporting guidance provided in the Preferred Reporting Items for Systematic Reviews and Meta-Analyses Protocols (PRISMA-P) statement (see checklist in Additional file 1) [16]. The protocol has been registered within the International Prospective Register of Systematic Reviews (PROSPERO) database (registration number CRD42020155964).

### Eligibility criteria

Studies will be selected according to the following criteria: population, intervention(s) of interest, control group, outcome(s), and context.

#### Population

All patient populations with hip and/or knee OA.

#### Intervention

Cost and outcomes for core treatments (education, exercise and dietary weight management) described in a dollar per outcome format (i.e., dollar per quality adjusted life year (QALY) or dollar per intermediate health outcome). We define education as any formal instruction about OA and self-management techniques. Exercise is defined as any activity requiring muscular contraction. Dietary weight management could include any intervention with the goal of caloric restriction.

#### Control

Any comparator that does not include surgical, pharmaceutical, or nutraceutical interventions where cost and outcomes are described in a dollar per outcome format (i.e., dollar per QALY or dollar per intermediate health outcome). Typically, economic evaluations use a comparator reflecting standard clinical practice, reported as “usual care,” although clinical trials may choose other comparators [12].

#### Outcomes

Full economic evaluations will compare both costs and health consequences of two or more interventions using a cost-utility analysis, cost-effectiveness analysis, cost-benefit analysis or cost-minimization analysis. Results in these publications will describe the incremental difference in cost and outcome estimates between the intervention and control groups. Results should be reported as cost-saving, incremental cost-utility ratio, incremental cost-effectiveness ratio, dominated by the control or a monetary value. The studies’ author will make a conclusion about of the cost-effectiveness of an intervention based on the reported outcome and the decision-makers’ willingness to pay for additional health benefits.

#### Context

Local practice patterns and health care system differences may influence the delivery, effectiveness, or resource demands associated with an intervention which requires an economic evaluation to be considered within the context of the health system where it is produced [17]**.** The studies’ author should provide rationale for the chosen perspective. This systematic review will include studies taking any perspective such as payer, health care sector, or societal perspective.

### Information sources

We will search the following electronic databases from inception to November 2019 without restriction in year or language: Medline, Embase, Cochrane Central Register of Controlled Trials (CENTRAL), National Health Services Economic Evaluation Database, and EconLit. Alerts will be set up in each database to notify DRM if additional publications meet our inclusion criteria until data extraction is complete. If a published study protocol matches the inclusion criteria but a subsequent paper has not been published the authors will be contacted to request preliminary unpublished data. The references of papers included in the systematic review will be hand searched to consider any relevant publications which may have been missed in the search strategy.

### Search strategy

Two researchers in collaboration with a content matter expert and librarian have developed a search strategy shown in Additional file 2. Three primary concepts were combined using the “OR” and “AND” Boolean operators. The primary concepts are shown in Table 1.

**Table 1:** Primary concepts and related Medical Subject Heading key words

Concept	#1	#2	#3
	Economic evaluation	Disease	Interventions
Key words	cost-benefit analysis, cost benefit analysis, benefits and costs, cost benefit, cost effectiveness, cost-utility analysis cost utility analysis, cost-effectiveness analysis, cost effectiveness analysis, cost-minimization analysis, cost minimization analysis, economic evaluation, marginal analysis	osteoarth* osteoarthritis, hip osteoarthritis, knee osteoarthritis, osteoarthrosis, ostoarthroses, degenerative arthritis	exercise, exercise therapy, aerobic exercise, exercise training, physical activity exercise rehabilitation, rehabilitation exercise, strength* or train* or exercis* or muscle train* or muscle strengthening or functional exercise* or flexibility train* or perturbation train* or proprioceptiv* or motor control or sensorimotor control or functional stability or dynamic stability or quality of movement or agility education, patient education, health education, community health education, diet, weight loss, diet therapy, calor* restriction, weight, weight reduction, weight management, diet modification, dietary modification, low-calorie, calor*, body mass

The Canadian Agency for Drug and Technology in Health has published search filters for MEDLINE and PubMed [18]. This search filter was modified to remove economic modeling terms and then combined with “Disease” and “Intervention” concepts using Boolean operators. Title, abstract, and author designated keywords will be searched for all relevant synonyms for economic evaluation, OA, exercise, education, and dietary weight management. Both reviewers (DM and AA) will execute the search strategy and import the citations to Covidence independently and in duplicate. Covidence is a web-based data management tool designed for health care evidence synthesis [19]. Authors have prepared a known list of economic evaluations that will be included in the systematic review. The quality of the search strategy will be determined by ensuring each publication on the list is present in at least one database.

### Study records

**Fig. 1:**
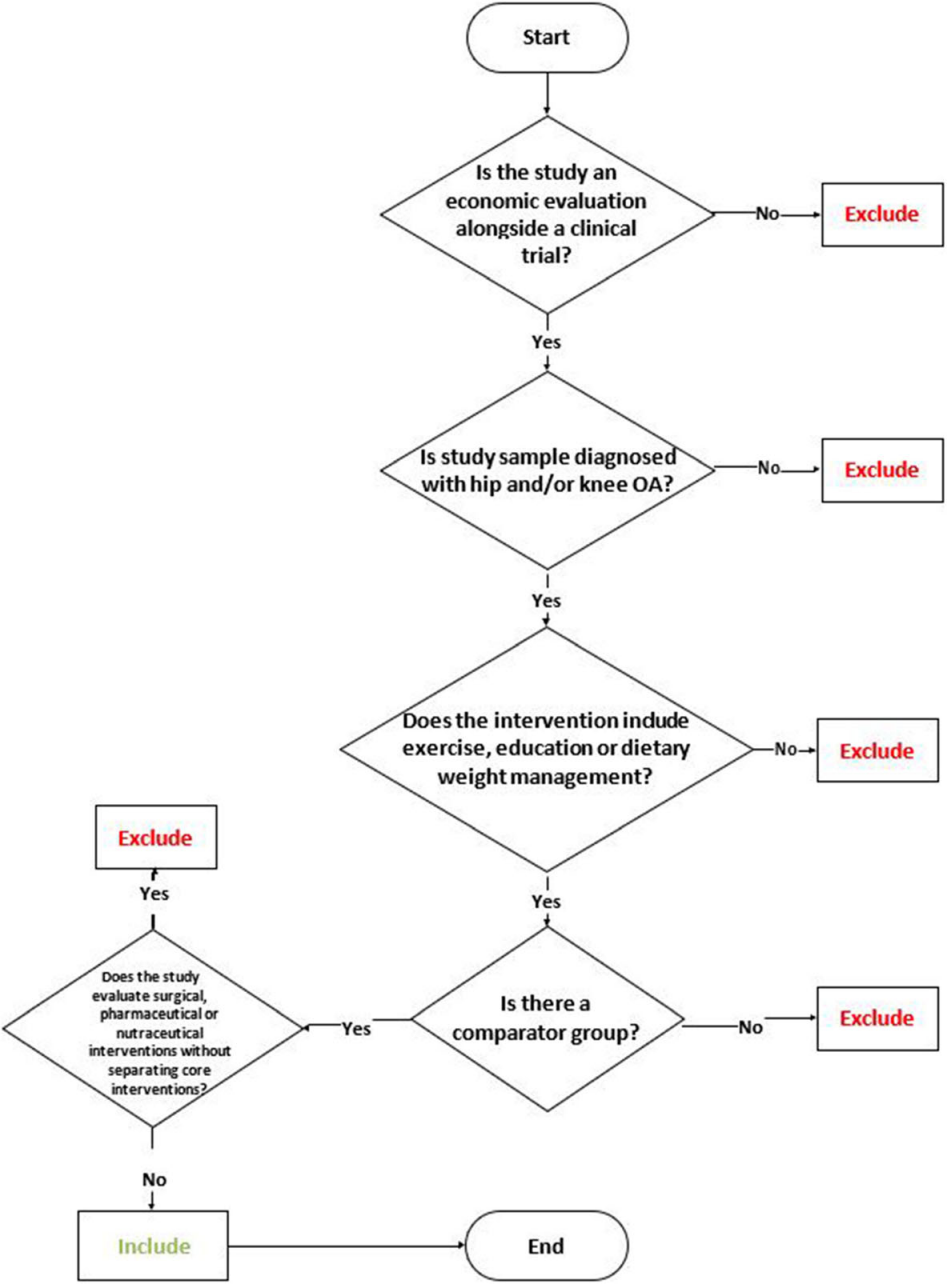
Selection process flow diagram

All citations from five databases will be exported to Covidence which automatically removes duplicate citations. Covidence will be used to screen titles and abstracts for inclusion and exclusion criteria. Reviewers will independently follow the screening process outlined in Fig. 1. Reviewers will include titles and abstracts with the following criteria:

1. Economic evaluations alongside randomized or nonrandomized clinical trials (cost-utility analysis, cost-effectiveness analysis, cost-benefit analysis, or cost-minimization analysis). Studies were considered a trial-based economic evaluation if patient-level costs and health outcomes were both collected during the clinical trial.
2. Population with any stage of knee and/or hip OA defined by the publication’s authors
3. Core interventions evaluated in isolation or combination (any type of exercise, education and dietary weight management) which may include additional adjunct therapies

Reviewers will remove titles and abstracts with the following exclusion criteria:

1. Trials evaluating surgical interventions unless the core intervention is evaluated separately
2. Trials evaluating pharmaceutical interventions unless the core intervention is evaluated separately
3. Trials evaluating nutraceutical interventions unless the core intervention is evaluated separately
4. Publications without a control group

Trials evaluating core treatments combined with surgical, pharmaceutical, or nutraceutical interventions are not included in this systematic review because the clinical effectiveness cannot be attributed solely to core treatments. Reviewers will meet to compare eligible papers before proceeding to full text review. Discrepancies will be adjudicated by the content expert. Full text review will proceed independently and in duplicate with the same process previously noted. A record of all items excluded during full text review will be kept with supporting rationale. A PRISMA flow diagram will be used to document study selection [16].

### Data items and outcomes

During full text review key elements from included papers will be extracted to an excel spreadsheet to populate a table of study characteristics and a table of key results. Key elements will align with the Consolidated Health Economic Evaluation Reporting Standards (CHEERS) checklist which is the most used reporting tool [11]. Extracted data will include author, year, target population, country, model of care, type of health care system, clinical trial study design, intervention, comparator, perspective, time horizon, type of analysis, health outcome measures, tariffs used to measure health outcome, itemized resource use, costing methodology for resource use, costing year, discount rate, mean QALY per person, mean costs per person, incremental cost per outcome, and the author’s cost-effectiveness recommendation (key outcome). Concluding an intervention is cost-effective depends on the perspective, country, and the decision-makers’ willingness to pay for additional health benefits. Two authors (DRM and AA) will split data extraction and the lead author (DM) will review all extracted data to ensure accuracy.

### Risk of bias in individual studies

As recommended by the Cochrane collaboration, this systematic review will evaluate risk of bias in trial-based economic evaluations by using the Consensus on Health Economic Criteria (CHEC) list [20, 21]. The CHEC checklist is a validated measure with 19 equally weighted characteristics designed to evaluate the methodological quality of economic evaluations conducted alongside clinical trials and observational studies [21]. Furthermore, a cumulative quality score will not be reported because the CHEC list does not include scoring criteria. Whether current instruments can discriminate between high- and low-quality economic evaluations is also up for debate [15, 22].

### Data synthesis

Study characteristics and key results will be synthesized in summary tables to descriptively explore heterogeneity. Currency from the study will be reported in the original units instead of converting to common units because study designs, intervention, controls, and health system characteristics limit comparability between cost-effectiveness results. To synthesize the results visually, all cost-utility results will be converted to 2019 US dollars using purchasing-power-parity exchange rates and plotted on a cost-utility plane. Caution should be exercised in comparing the results since these estimates do not account for different comparators and health care system characteristics.

Methods for pooling cost-effectiveness estimates do not currently exist [20]. Economic evaluations often produce heterogeneous results because the costs of providing care differ greatly between countries. Due to this variability, a meta-analysis will not be attempted.

Provided there are enough papers, a subgroup analysis will be attempted to investigate whether certain study characteristics are more likely to produce a cost-effective recommendation. The following subgroup analyses will be attempted:

1. Randomized versus nonrandomized study designs
2. Treatment received (exercise, education, diet, or combination of treatments)
3. Model of care (single professional, integrated specialty service, or multidisciplinary team)
4. Type of health care system (determined by country)
5. Severity of OA included in the sample population

## Discussion

To support the implementation of OAMPs, we will provide a current overview of published literature evaluating the cost-effectiveness of core treatments to manage hip and knee OA. Synthesizing economic evaluations for core treatments will describe progress in the research field and enable analysts to assess transferability of the results to local decision problems. This work may identify current gaps in the literature which will provide direction for future research. Subgroup analysis will attempt to identify common characteristics between studies, health systems, and models of care which affect the cost-effectiveness results for core treatments. Lastly, this review will allow the authors to explore the tradeoffs between resource allocation and health outcomes for the management of OA using core treatments.

There is substantial difference between the methodologies to conduct economic evaluations using modeling or trial-based economic evaluations [6]. As recommended by previous authors, this systematic review will focus on one methodology [14]. Trial-based economic evaluations are more popular in nonsurgical and nonpharmacological economic research [9]. This literature synthesis will also enable future transferability assessments [23]. Our analysis will differ from Pinto et al. as they used the Quality of Health Economic Analyses instrument although it is recommended as a quality assessment tool for model-based economic evaluations [7, 8, 15]. The CHEC list is the preferred quality assessment tool for systematic reviews of economic evaluations alongside clinical trials [8, 15]. A systematic review of economic evaluation quality assessment tools found the CHEC list had strong criterion validity and inter-rater reliability [15]. The publication of reporting standards and methodological guidelines in the past decade has progressed in the health economics research field [9–13]. This review will attempt to understand whether methodological quality has improved in economic evaluations of OA interventions since reporting guidelines were published.

Ample research shows the clinical effectiveness of core treatments for OA [2, 24, 25]. However, the cost-effectiveness of these interventions remains unclear [7]. Cost-effectiveness research plays an important role for health services decision-making by describing the tradeoffs between resources consumed for health outcomes gained. In a budget-constrained health care system, economic evaluations are used to inform decision-making for the adoption of new treatments and technologies [6]. As research shifts towards the implementation of OAMPs, a current systematic review of economic evaluations is warranted. This systematic review will synthesize the economic literature, explore gaps in the research field, and support future research of recommended treatments for OA.

## Supplementary information

**Additional file 1.** Mazzei et al PRISMA-P checklist.

**Additional file 2.** Mazzei et al Systematic Review Search Strategy.

## Data Availability

Not applicable.

## Abbreviations

OA: Osteoarthritis
OAMP: Osteoarthritis management program
CENTRAL: Cochrane Central Register of Controlled Trials
EMBASE: Excerpta Medica Database
MEDLINE: Medical Literature Analysis and Retrieval System Online
PRISMA: Preferred Reporting Items for Systematic Reviews and Meta-Analyses
QALY: Quality adjusted life year

## References

[1] Cross M, Smith E, Hoy D, Nolte S, Ackerman I, Fransen M (2014). The global burden of hip and knee osteoarthritis: estimates from the global burden of disease 2010 study. Ann Rheum Dis.

[2] Bannuru RR, Osani MC, Vaysbrot EE, Arden NK, Bennell K, Bierma-Zeinstra SMA (2019). OARSI guidelines for the non-surgical management of knee, hip, and polyarticular osteoarthritis. Osteoarthr Cartil.

[3] Hagen KB, Smedslund G, Osteras N, Jamtvedt G (2016). Quality of community based osteoarthritis care: a systematic review. Arthritis Care Res.

[4] Allen KD, Choong PF, Davis AM, Dowsey MM, Dziedzic KS, Emery C (2016). Osteoarthritis: Models for appropriate care across the disease continuum. Best Pract Res Clin Rheumatol.

[5] Eyles JP, Hunter DJ, Bennel KL, Dziedzic KS, Hinman RS, Van der Esch M (2019). Priorities for the effective implementation of osteoarthritis management programs: an OARSI international consensus exercise. Osteoarthr Cartil.

[6] Torrance GW, Drummond MF, Sculpher MJ, Claxton K, Stoddart GL (2015). Methods for the economic evaluation of health care programmes.

[7] Pinto D, Robertson MC, Hansen P, Abbott JH (2012). Cost-effectiveness of nonpharmacologic, nonsurgical interventions for hip and/or knee osteoarthritis: systematic review. Value Health.

[8] Wijnen BFM, Van Mastrigt GAPG, Redekop WK, Majoie HJM, DeKinderen RJA, Evers SMAA (2016). How to prepare a systematic review of economic evaluations for informing evidence-based healthcare decisions: data extraction, risk of bias, and transferability (part 3/3). Ex Rev Pharmacoeconomics Outcomes Res.

[9] Hiligsmann M, Cooper C, Arden N, Boers M, Branco J, Brandi ML (2013). Health economics in the field of osteoarthritis: an expert’s consensus paper from the European Society of Economic Aspects of Osteoperosis and Osteoarthritis (ESCEO). Sem Arth Rhem.

[10] Hiligsmann M, Cooper C, Arden N, Boers M, Branco J, Brandi ML (2014). A reference case for economic evaluations in osteoarthritis: an expert’s consensus paper from the European Society of Economic Aspects of Osteoperosis and Osteoarthritis (ESCEO). Sem Arth Rhem.

[11] Husereau D, Drummond M, Petrou S (2013). ISPOR Health Economic Evaluation Publication Guidelines-CHEERS Good Reporting Practices Task Force. Consolidated Health Economic Evaluation Reporting Standards (CHEERS)—explanation and elaboration: a report of the ISPOR Health Economic Evaluation Publication Guidelines Good Reporting Practices Task Force. Value Health.

[12] Ramsey SD, Willke RJ, Glick H, Reed SD, Augustovski F, Jonsson B (2015). Cost-effectiveness analysis alongside clinical trials II—an ISPOR Good Research Practices Task Force report. Value Health.

[13] Guidelines for the economic evaluation of health technologies: Canada. 4th. Ottawa: CADTH; 2017.

[14] Anderson R (2010). Systematic reviews of economic evaluations: utility or futility?. Health Econ.

[15] Walker DG, Wilson RF, Sharma R, Bridges J, Niessen L, Bass EB, Frick K (2012). Best practices for conducting economic evaluations in health care: a systematic review of quality assessment tools. Methods Research Report. The Johns Hopkins University Evidence-based Practice Center.

[16] Moher D, Shamseer L, Clarke M, Ghersi D, Liberati A, Petticrew M (2015). PRISMA-P Group. Preferred reporting items for the sytstematic review and meta-analysis protocols (PRISMA-P) 2015 Statement. Systematic Rev.

[17] Gomersall JS, Jadotte YT, Xue Y, Lockwood S, Riddle D, Preda A (2015). Conducting systematic reviews of economic evaluations. Int J Evid Based Healthc.

[18] Strings attached: CADTH database search filters. Ottawa: CADTH; 2016. [cited 2019 Oct 10]. Available from: https://www.cadth.ca/resources/finding-evidence.

[19] Covidence: Better systematic review management. Melbourne: Covidence. [cited 2019 Oct 10]. Available from: https://www.covidence.org/home.

[20] Higgins J.P.T, Green S (editors). Cochrane handbook for systematic reviews of interventions version 5.1.0 [updated March 2011]. The Cochrane Collaboration. 2011. Available from: www.handbook.cochrane.org.

[21] Evers S, Goossens M, de Vet H, van Tulder M, Ament A (2005). Criteria list for assessment of methodological quality of economic evaluations: consensus on health economic criteria. Int J Technol Assess Health Care.

[22] Frederix GWJ (2019). Check Your Checklist: The danger of over- and underestimating the quality of economic evaluations. Pharmaco Economics Open.

[23] Welte R, Feenstra T, Jager H, Leidl R (2004). A decision chart for assessing and improving the transferability of economic evaluation results between countries. Pharmacoeconomics..

[24] Fransen M, McConnell S, Harmer AR, Van der Esch M, Simic M, Bennell KL. Exercise for osteoarthritis of the knee. Cochrane Database Syst Rev. 2015;1. 10.1002/14651858.CD004376.pub3. 10.1002/14651858.CD004376.pub3 PMC10094004 25569281

[25] Fransen M, McConnell S, Hernandez-Molina G, Reichenbach S. Exercise for osteoarthritis of the hip. Cochrane Database Syst Rev. 2014;4. 10.1002/14651858.CD007912.pub2. 10.1002/14651858.CD007912.pub2 PMC10898220 24756895

