# Supplementary material for: Systematic review of economic evaluations investigating education, exercise, and dietary weight management to manage hip and knee osteoarthritis: protocol
Source: Syst Rev. 2020 Oct 6;9:229. doi: 10.1186/s13643-020-01492-6 (PMC7542349; doi:10.1186/s13643-020-01492-6)
Supplement: Supplementary file 1 — Additional file 1. Mazzei et al PRISMA-P checklist. [file 13643_2020_1492_MOESM1_ESM.docx]

**PRISMA-P checklist.**

| Section and topic | Item No | Checklist item Where in the protocol (Page) |
| --- | --- | --- |
| ADMINISTRATIVE INFORMATION | | |
| Title: |  |  |
| Identification | 1a | Identify the report as a protocol of a systematic review Page 1 |
| Update | 1b | If the protocol is for an update of a previous systematic review,  identify as such Not Applicable |
| Registration | 2 | If registered, provide the name of the registry (such as PROSPERO)  and registration number Page 2 |
| Authors: |  |  |
| Contact | 3a | Provide name, institutional affiliation, e-mail address of all protocol authors; Page 1  provide physical mailing address of corresponding author |
| Contributions | 3b | Describe contributions of protocol authors and identify the guarantor of Page 15  the review |
| Amendments | 4 | If the protocol represents an amendment of a previously completed or Not Applicable  published protocol, identify as such and list changes; otherwise,  state plan for documenting important protocol amendments |
| Support: |  |  |
| Sources | 5a | Indicate sources of financial or other support for the review Page 15 |
| Sponsor | 5b | Provide name for the review funder and/or sponsor Not Applicable |
| Role of sponsor or funder | 5c | Describe roles of funder(s), sponsor(s), and/or institution(s), Not Applicable  if any, in developing the protocol |
| INTRODUCTION | | |
| Rationale | 6 | Describe the rationale for the review in the context of what is Page 3  already known |
| Objectives | 7 | Provide an explicit statement of the question(s) the review will address Pages 5  with reference to participants, interventions, comparators, and outcomes  (PICO) |
| METHODS | | |
| Eligibility criteria | 8 | Specify the study characteristics (such as PICO, study design, setting, time frame) Pages 5-6  and report characteristics (such as years considered, language, publication status)  to be used as criteria for eligibility for the review |
| Information sources | 9 | Describe all intended information sources (such as electronic databases, contact Page 7  with study authors, trial registers or other grey literature sources)  with planned dates of coverage |
| Search strategy | 10 | Present draft of search strategy to be used for at least one electronic database, Pages 7-8  including planned limits, such that it could be repeated |
| Study records: |  |  |
| Data management | 11a | Describe the mechanism(s) that will be used to manage records and data Page 8-9  throughout the review |
| Selection process | 11b | State the process that will be used for selecting studies (such as two Page 7-10  independent reviewers) through each phase of the review (that is, screening,  eligibility and inclusion in meta-analysis) |
| Data collection process | 11c | Describe planned method of extracting data from reports (such as piloting forms, Page 10  done independently, in duplicate), any processes for obtaining and confirming  data from investigators |
| Data items | 12 | List and define all variables for which data will be sought (such as PICO items, Page 9-10  funding sources), any pre-planned data assumptions and simplifications |
| Outcomes and prioritization | 13 | List and define all outcomes for which data will be sought, including prioritization Page 10  of main and additional outcomes, with rationale |
| Risk of bias in individual studies | 14 | Describe anticipated methods for assessing risk of bias of individual studies, Page 10-11  including whether this will be done at the outcome or study level, or both;  state how this information will be used in data synthesis |
| Data synthesis | 15a | Describe criteria under which study data will be quantitatively synthesised Pages 11-12 |
|  | 15b | If data are appropriate for quantitative synthesis, describe planned Not Applicable  summary measures, methods of handling data and methods of combining  data from studies, including any planned exploration of consistency  (such as I^2^, Kendall’s τ) |
|  | 15c | Describe any proposed additional analyses (such as sensitivity Page 11-12  or subgroup analyses, meta-regression) |
|  | 15d | If quantitative synthesis is not appropriate, describe the type of Not Applicable  summary planned |
| Meta-bias(es) | 16 | Specify any planned assessment of meta-bias(es) (such as publication bias Not Applicable  across studies, selective reporting within studies) |
| Confidence in cumulative evidence | 17 | Describe how the strength of the body of evidence will be assessed Page 10-11  (such as GRADE) |

*From: Shamseer L, Moher D, Clarke M, Ghersi D, Liberati A, Petticrew M, Shekelle P, Stewart L, PRISMA-P Group. Preferred reporting items for systematic review and meta-analysis protocols (PRISMA-P) 2015: elaboration and explanation. BMJ. 2015 Jan 2;349(jan02 1):g7647.*
