# Supplementary material for: Systematic review of economic evaluations investigating education, exercise, and dietary weight management to manage hip and knee osteoarthritis: protocol
Source: Syst Rev. 2020 Oct 6;9:229. doi: 10.1186/s13643-020-01492-6 (PMC7542349; doi:10.1186/s13643-020-01492-6)
Supplement: Supplementary file 2 — Additional file 2. Mazzei et al Systematic Review Search Strategy. [file 13643_2020_1492_MOESM2_ESM.pdf]

Systematic Review Search Criteria

Medline

1. Economics/
2. Cost/
3. Economics, Nursing/
4. Economics, Medical/
5. Economics, Pharmaceutical/
6. exp Economics, Hospital/
7. exp "Fees and Charges"/
8. exp Budgets/
9. budget*.ti,ab,kf.
10. (economic* or cost or costs or costly or costing or price or prices or pricing or pharmacoeconomic* or pharmaco-economic* or expenditure or expenditures or expense or expenses or financial or finance or finances or financed).ti,kf.
11. (economic* or cost or costs or costly or costing or price or prices or pricing or pharmacoeconomic* or pharmaco-economic* or expenditure or expenditures or expense or expenses or financial or finance or finances or financed).ab. /freq=2
12. (cost* adj2 (effective* or utilit* or benefit* or minimi* or analy* or outcome or outcomes)).ab,kf.
13. (value adj2 (money or monetary)).ti,ab,kf.
14. or/1-13
15. Hip Osteoarthritis/ or Knee Osteoarthritis/
16. (osteoarthriti* or osteoarthros* or arthrit* or arthros* or degenerative or joint disorder* or joint disease* or knee oa or hip oa) adj3 (knee* or hip*)).ti,ab,kf
17. 15 or 16
18. Exercise Therapy/ or Exercise/
19. (physiother* or exercis* or therap* or program* or structured or targeted or semi structured or supervised or self management).ti,ab,kf.
20. (strength* or train* or exercis* or muscle train* or muscle strengthening or functional exercise* or flexibility train* or perturbation train* or proprioceptiv* or motor control or sensorimotor control or functional stability or dynamic stability or quality of movement or agility).ti,ab,kf.

21. exp Patient Education/ or exp Education/ or exp Health Education/ or education.tw,kf.
22. educat*.ti,ab,kf.
23. exp Diet/ or Weight Loss/ or Diet Therapy/
24. (diet or diet therapy or weight or weight management or weight loss or calor* or calor* restriction or body mass).ti,ab,kf.
25. 18 or 19 or 20 or 21 or 22 or 23 or 24
26. 14 and 17 and 25

Embase

1. Economics/
2. Cost/
3. Exp Health Economics/
4. Budget/
5. budget*.ti,ab,kw.
6. (economic* or cost or costs or costly or costing or price or prices or pricing or pharmacoeconomic* or pharmaco-economic* or expenditure or expenditures or expense or expenses or financial or finance or finances or financed).ti,kw.
7. (economic* or cost or costs or costly or costing or price or prices or pricing or pharmacoeconomic* or pharmaco-economic* or expenditure or expenditures or expense or expenses or financial or finance or finances or financed).ab. /freq=2
8. (cost* adj2 (effective* or utilit* or benefit* or minimi* or analy* or outcome or outcomes)).ab,kw.
9. (value adj2 (money or monetary)).ti,ab,kw.
10. 1 or 2 or 3 or 4 or 5 or 6 or 7 or 8 or 9
11. Hip Osteoarthritis/ or Knee Osteoarthritis/
12. (osteoarthriti* or osteoarthros* or arthrit* or arthros* or degenerative or joint disorder* or joint disease* or knee oa or hip oa) adj3 (knee* or hip*)).ti,ab,kw
13. 11 or 12
14. Exercise Therapy/ or Exercise/
15. (physiother* or exercis* or therap* or program* or structured or targeted or semi structured or supervised or self management).ti,ab,kw.

16. (strength* or train* or exercis* or muscle train* or muscle strengthening or functional exercise* or flexibility train* or perturbation train* or proprioceptiv* or motor control or sensorimotor control or functional stability or dynamic stability or quality of movement or agility).ti,ab,kw.
17. exp Patient Education/ or exp Education/ or exp Health Education/ or education.tw,Kw.
18. educat*.ti,ab,kw.
19. Diet/ or Weight Loss/ or Diet Therapy/
20. (diet or diet therapy or weight or weight management or weight loss or calor* or calor* restriction or body mass).ti,ab,kw.
21. 14 or 15 or 16 or 17 or 18 or 19 or 20
22. 10 and 13 and 21

Cochrane Central Register of Controlled Trials (CENTRAL)

1. Exp Economics/
2. Economics, Pharmaceutical/ or Economics, Behavioral/ or Economics, Medical/ or Economics/ or Economics, Hospital/ or Economics, Nursing/
3. (Fees and Charges).mp. [mp=title, original title, abstract, mesh headings, heading words, keyword]
4. Budgets/
5. budget*.ti,tw,kw.
6. (economic* or cost or costs or costly or costing or price or prices or pricing or pharmacoeconomic* or pharmaco-economic* or expenditure or expenditures or expense or expenses or financial or finance or finances or financed).ti,ab,kw.
7. (cost* adj2 (effective* or utilit* or benefit* or minimi* or analy* or outcome or outcomes)).ti,ab,kw.
8. (value adj2 (money or monetary)).ti,ab,kw.
9. 1 or 2 or 3 or 4 or 5 or 6 or 7 or 8
10. exp Osteoarthritis/ or exp Osteoarthritis,Hip/ or exp Osteoarthritis,Knee/
11. (osteoarthriti* or osteoarthros* or arthrit* or arthros* or degenerative or joint disorder* or joint disease* or knee oa or hip oa) adj3 (knee* or hip*)).ti,ab,kw.
12. 10 or 11
13. Exercise Therapy/ or Exercise/

14. (physiother* or exercis* or therap* or program* or structured or targeted or semi structured or supervised or self management).ti,ab,kw.
15. (strength* or train* or exercis* or muscle train* or muscle strengthening or functional exercise* or flexibility train* or perturbation train* or proprioceptiv* or motor control or sensorimotor control or functional stability or dynamic stability or quality of movement or agility).ti,ab,kw.
16. Exp Patient Education as Topic/ or exp Education/ or exp Health Education/ or education.tw,kw.
17. educat*.ti,ab,kw.
18. Diet/ or Weight Loss/ or Diet Therapy/
19. (diet or diet therapy or weight or weight management or weight loss or calor* or calor* restriction or body mass).ti,ab,kw.
20. 13 or 14 or 15 or 16 or 17 or 18 or 19
21. 9 and 12 and 20

National Health Services Economic Evaluation Database

22. Economics, Pharmaceutical/ or Economics, Behavioral/ or Economics, Medical/ or Economics/ or Economics, Hospital/ or Economics, Nursing/
23. (Fees and Charges).mp. [mp=title,text, subject heading word]
24. Budgets/
25. budget*.ti,tw,sh.
26. (economic* or cost or costs or costly or costing or price or prices or pricing or pharmacoeconomic* or pharmaco-economic* or expenditure or expenditures or expense or expenses or financial or finance or finances or financed) .ti,tw,sh.
27. (cost* adj2 (effective* or utilit* or benefit* or minimi* or analy* or outcome or outcomes)) .ti,tw,sh.
28. (value adj2 (money or monetary)) .ti,tw,sh.
29. 1 or 2 or 3 or 4 or 5 or 6 or 7
30. exp Osteoarthritis/ or exp Osteoarthritis,Hip/ or exp Osteoarthritis,Knee/
31. (osteoarthriti* or osteoarthros* or arthrit* or arthros* or degenerative or joint disorder* or joint disease* or knee oa or hip oa) adj3 (knee* or hip*)). .ti,tw,sh.
32. 9 or 10

33. Exercise Therapy/ or Exercise/
34. (physiother* or exercis* or therap* or program* or structured or targeted or semi structured or supervised or self management).ti,tw,sh.
35. (strength* or train* or exercis* or muscle train* or muscle strengthening or functional exercise* or flexibility train* or perturbation train* or proprioceptiv* or motor control or sensorimotor control or functional stability or dynamic stability or quality of movement or agility) .ti,tw,sh.
36. exp Education/ or exp Health Education/ or education.tw,sh.
37. educat* .ti,tw,sh.
38. Diet/ or Weight Loss/ or Diet Therapy/
39. (diet or diet therapy or weight or weight management or weight loss or calor* or calor* restriction or body mass).ti,tw,sh.
40. 12 or 13 or 14 or 15 or 16 or 17 or 18
41. 8 and 11 and 19

EconLit

#	Query	Limiters/Expanders	Last Run Via
S39	(S17 AND S23 AND S38)	Search modes - Find all my search terms	Interface - EBSCOhost Research Databases<br>Search Screen - Advanced Search<br>Database - EconLit
S38	S24 OR S25 OR S26 OR S27 OR S28 OR S29 OR S30 OR S31 OR S32 OR S33 OR S34 OR S35 OR S36 OR S37	Search modes - Find all my search terms	Interface - EBSCOhost Research Databases<br>Search Screen - Advanced Search<br>Database - EconLit

S37	AB (diet or diet therapy or weight or weight management or weight loss or calor* or calor* restriction or body mass)	Search modes - Find all my search terms	Interface - EBSCOhost Research Databases<br>Search Screen - Advanced Search<br>Database - EconLit
S36	TI (diet or diet therapy or weight or weight management or weight loss or calor* or calor* restriction or body mass)	Search modes - Find all my search terms	Interface - EBSCOhost Research Databases<br>Search Screen - Advanced Search<br>Database - EconLit
S35	MH Caloric Restriction	Search modes - Find all my search terms	Interface - EBSCOhost Research Databases<br>Search Screen - Advanced Search<br>Database - EconLit
S34	MH (diet therapy or dietetic therapy or diet or nutrition interventions)	Search modes - Find all my search terms	Interface - EBSCOhost Research Databases<br>Search Screen - Advanced Search<br>Database - EconLit
S33	MH (weight loss or weight reduction or lose weight or obesity or overweight or weight management)	Search modes - Find all my search terms	Interface - EBSCOhost Research Databases<br>Search Screen - Advanced Search<br>Database - EconLit
S32	MH (diet or nutrition or food habit or eating habit or lifestyle)	Search modes - Find all my search terms	Interface - EBSCOhost Research Databases

			Search Screen - Advanced Search<br>Database - EconLit
S31	AB (educat*)	Search modes - Find all my search terms	Interface - EBSCOhost Research Databases<br>Search Screen - Advanced Search<br>Database - EconLit
S30	TI (educat*)	Search modes - Find all my search terms	Interface - EBSCOhost Research Databases<br>Search Screen - Advanced Search<br>Database - EconLit
S29	MH (Patient Education or Education or Health Education)	Search modes - Find all my search terms	Interface - EBSCOhost Research Databases<br>Search Screen - Advanced Search<br>Database - EconLit
S28	AB (strength* or train* or exercis* or muscle train* or muscle strengthening or functional exercise* or flexibility train* or perturbation train* or proprioceptiv* or motor control or sensorimotor control or functional stability or dynamic stability or quality of movement or agility)	Search modes - Find all my search terms	Interface - EBSCOhost Research Databases<br>Search Screen - Advanced Search<br>Database - EconLit
S27	TI (strength* or train* or exercis* or muscle train* or muscle strengthening or functional exercise* or flexibility train* or perturbation train* or proprioceptiv* or motor	Search modes - Find all my search terms	Interface - EBSCOhost Research Databases<br>Search Screen - Advanced Search<br>Database - EconLit

	control or sensorimotor control or functional stability or dynamic stability or quality of movement or agility)		
S26	AB (physiother* or exercis* or therap* or program* or structured or targeted or semi structured or supervised or self management)	Search modes - Find all my search terms	Interface - EBSCOhost Research Databases<br>Search Screen - Advanced Search<br>Database - EconLit
S25	TI (physiother* or exercis* or therap* or program* or structured or targeted or semi structured or supervised or self management)	Search modes - Find all my search terms	Interface - EBSCOhost Research Databases<br>Search Screen - Advanced Search<br>Database - EconLit
S24	MH (Exercise Therapy OR Exercise)	Search modes - Find all my search terms	Interface - EBSCOhost Research Databases<br>Search Screen - Advanced Search<br>Database - EconLit
S23	S18 OR S19 OR S20 OR S21 OR S22	Search modes - Find all my search terms	Interface - EBSCOhost Research Databases<br>Search Screen - Advanced Search<br>Database - EconLit
S22	AB (osteoarthriti* or osteoarthros* or arthrit* or arthros* or degenerative or joint disorder* or joint disease* or knee oa or hip oa)	Search modes - Find all my search terms	Interface - EBSCOhost Research Databases<br>Search Screen - Advanced Search<br>Database - EconLit

S21	TI (osteoarthriti* or osteoarthros* or arthrit* or arthros* or degenerative or joint disorder* or joint disease* or knee oa or hip oa)	Search modes - Find all my search terms	Interface - EBSCOhost Research Databases<br>Search Screen - Advanced Search<br>Database - EconLit
S20	MH (Osteoarthritis, Knee)	Search modes - Find all my search terms	Interface - EBSCOhost Research Databases<br>Search Screen - Advanced Search<br>Database - EconLit
S19	MH (Osteoarthritis, Hip)	Search modes - Find all my search terms	Interface - EBSCOhost Research Databases<br>Search Screen - Advanced Search<br>Database - EconLit
S18	MH (Osteoarthritis)	Search modes - Find all my search terms	Interface - EBSCOhost Research Databases<br>Search Screen - Advanced Search<br>Database - EconLit
S17	S1 OR S2 OR S3 OR S4 OR S5 OR S6 OR S7 OR S8 OR S9 OR S10 OR S11 OR S12 OR S13 OR S14 OR S15 OR S16	Search modes - Find all my search terms	Interface - EBSCOhost Research Databases<br>Search Screen - Advanced Search<br>Database - EconLit
S16	AB (value money or value monetary)	Search modes - Find all my search terms	Interface - EBSCOhost Research Databases

			Search Screen - Advanced Search<br>Database - EconLit
S15	TI (value money or value monetary)	Search modes - Find all my search terms	Interface - EBSCOhost Research Databases<br>Search Screen - Advanced Search<br>Database - EconLit
S14	AB (cost* effective* or cost* utilit* or cost* benefit* or cost* minimi* or cost* analy* or cost* outcome or cost* outcomes)	Search modes - Find all my search terms	Interface - EBSCOhost Research Databases<br>Search Screen - Advanced Search<br>Database - EconLit
S13	TI (cost* effective* or cost* utilit* or cost* benefit* or cost* minimi* or cost* analy* or cost* outcome or cost* outcomes)	Search modes - Find all my search terms	Interface - EBSCOhost Research Databases<br>Search Screen - Advanced Search<br>Database - EconLit
S12	(economic* or cost or costs or costly or costing or price or prices or pricing or pharmacoeconomic* or pharmaco-economic* or expenditure or expenditures or expense or expenses or financial or finance or finances or financed).ab. /freq=2	Search modes - SmartText Searching	Interface - EBSCOhost Research Databases<br>Search Screen - Advanced Search<br>Database - EconLit
S11	TI (economic* or cost or costs or costly or costing or price or prices or pricing or pharmacoeconomic* or pharmaco-economic* or expenditure or expenditures or expense or	Search modes - Find all my search terms	Interface - EBSCOhost Research Databases<br>Search Screen - Advanced Search<br>Database - EconLit

	expenses or financial or finance or finances or financed).ab. /freq=2		
S10	AB (economic* or cost or costs or costly or costing or price or prices or pricing or pharmacoeconomic* or pharmaco-economic* or expenditure or expenditures or expense or expenses or financial or finance or finances or financed)	Search modes - Find all my search terms	Interface - EBSCOhost Research Databases<br>Search Screen - Advanced Search<br>Database - EconLit
S9	TI (economic* or cost or costs or costly or costing or price or prices or pricing or pharmacoeconomic* or pharmaco-economic* or expenditure or expenditures or expense or expenses or financial or finance or finances or financed)	Search modes - Find all my search terms	Interface - EBSCOhost Research Databases<br>Search Screen - Advanced Search<br>Database - EconLit
S8	MH (Budgets)	Search modes - Find all my search terms	Interface - EBSCOhost Research Databases<br>Search Screen - Advanced Search<br>Database - EconLit
S7	MH Fees and Charges	Search modes - Find all my search terms	Interface - EBSCOhost Research Databases<br>Search Screen - Advanced Search<br>Database - EconLit
S6	MH Economics, Hospital	Search modes - Find all my search terms	Interface - EBSCOhost Research Databases<br>Search Screen - Advanced Search<br>Database - EconLit

S5	MH Economics, Pharmaceutical	Search modes - Find all my search terms	Interface - EBSCOhost Research Databases<br>Search Screen - Advanced Search<br>Database - EconLit
S4	MH Economics, Medical	Search modes - Find all my search terms	Interface - EBSCOhost Research Databases<br>Search Screen - Advanced Search<br>Database - EconLit
S3	MH Economics, Nursing	Search modes - Find all my search terms	Interface - EBSCOhost Research Databases<br>Search Screen - Advanced Search<br>Database - EconLit
S2	MH Costs and Cost Analysis	Search modes - Find all my search terms	Interface - EBSCOhost Research Databases<br>Search Screen - Advanced Search<br>Database - EconLit
S1	MH economics	Search modes - Find all my search terms	Interface - EBSCOhost Research Databases<br>Search Screen - Advanced Search<br>Database - EconLit
